# Primary non-Hodgkin’s lymphoma of the prostate with intractable hematuria: A case report and review of the literature

**DOI:** 10.3892/ol.2014.2829

**Published:** 2014-12-24

**Authors:** SHANBIAO HU, YINHUAI WANG, LUOYAN YANG, LU YI, YEQI NIAN

**Affiliations:** Department of Urology, Second Xiangya Hospital, Central South University, Changsha, Hunan, P.R. China

**Keywords:** primary non-Hodgkin’s lymphoma, prostate, embolization, R-CHOP

## Abstract

Cases of primary non-Hodgkin’s lymphoma of the prostate are globally rare. The present study reports a case of prostatic diffuse large B-cell lymphoma (DLBCL) with intractable hematuria in a 75-year-old male. The patient presented with difficulties in urination and gross hematuria. A prostate biopsy was performed immediately, followed by conservative treatment for bleeding. A bilateral iliac arteriography and chemoembolization were then performed as emergency procedures under local anesthesia due to significant bleeding and a sharply decreased blood pressure, indicating the failure of the conservative treatment. Consequently, the bleeding was effectively controlled. Pathological examination of the prostate biopsy confirmed the presence of a DLBCL of non-germinal center B-cell origin. Immunohistochemical examination demonstrated cluster of differentiation (CD)20(++), CD3(+), leukocyte common antigen(+++), B-cell lymphoma-2(+) and prostate-specific antigen(−) results. Due to the poor general condition and low hemoglobin levels of the patient, a low-dose Rituximab plus cyclophosphamide, doxorubicin, vincristine and prednisone (R-CHOP) chemotherapy regimen was administered. Subsequent to three courses of chemotherapy, the patient achieved complete remission. In conclusion, combining R-CHOP and bilateral selective iliac arterial chemoembolization could be a safe and effective way to treat patients with non-Hodgkin’s lymphoma of the prostate and intractable hematuria.

## Introduction

Primary malignant lymphomas of the prostate are rare, and the majority are reported as single cases. The tumors account for 0.09% of all prostate neoplasms and 0.1% of all non-Hodgkin’s lymphomas (NHLs) (1). Non-Hodgkin’s lymphoma is the major histological subtype of primary Hodgkin’s lymphoma (PHL) and diffuse large B-cell lymphoma (DLBCL) is the most common type of NHL. As primary lymphoma of the prostate is rare, at present there is no consensus regarding its optimal management, however, current treatment modalities include chemotherapy, radiotherapy and radical prostatectomy. Advanced pelvic urological malignancies are the most common cause of intractable haematuria (2–4). Blood loss as a result of intractable haematuria is often severe and the condition of the patient is generally poor and thus, to avoid the additional risk associated with surgical intervention, selective transarterial embolization of the internal iliac arteries has been found to be safe and highly effective for patients with severe bladder and prostatic haemorrhage (2,4). The current study presents the case of a 75-year-old male with prostatic DLBCL and intractable hematuria, which was successfully controlled by a low dose of rituximab combined with cyclophosphamide, doxorubicin, vincristine and prednisone (R-CHOP), and bilateral selective iliac arterial chemoembolization. Written informed consent was obtained from the patient.

## Case report

A 75-year-old male was admitted to the Department of Urology in the Second Xiangya Hospital (Central South University, Changsha, Hunan, China) due to difficulty in urinating, with a one-year history of urinary obstruction and a 10-day history of gross hematuria. A physical examination revealed hypertrophy of the prostate, and disappearance of the median sulcus. There was no palpable enlargement of the superficial lymph nodes and no hepatosplenomegaly.

Laboratory findings, including blood count, and liver and renal function, were all normal. Chest X-rays revealed no abnormalities. Computed tomography (CT) showed no evidence of distant metastasis to the lungs or abdomen. The concentrations of tumor markers, including α-fetoprotein, carcinoembryonic antigen and prostate-specific antigen (PSA), were within normal limits. The level of serum lactate dehydrogenase (LDH) was elevated to 375.4 U/l (normal range, 104.0–245.0 U/l) and the concentration of β2-microglobulin was 3.89 mg/l (normal range, 0.80–2.40 mg/l).

**Figure 1:**
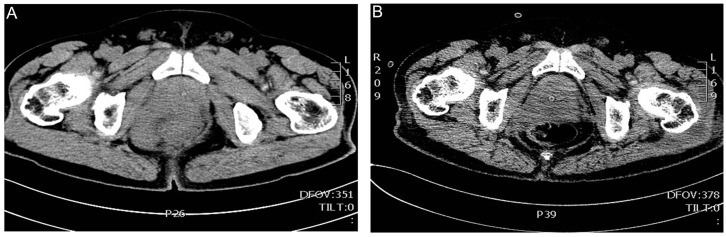
Computed tomography (CT) scans prior to and following treatment. (A) CT scan clearly showing a prostatic space-occupying lesion with a maximum intersecting surface 8.3×7.1 cm in size, fuzzy boundaries with the rectum and an absence of the bladder seminal vesicle angle prior to treatment. (B) Following treatment, the prostate was almost back to normal, with relatively clear boundaries with the rectum.

**Figure 2:**
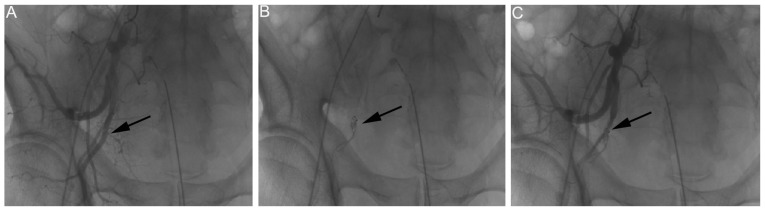
Embolization procedure at the point of the bleeding (arrows). (A) Angiography revealing bleeding. (B) Embolization. (C) No further bleeding following embolization.

**Figure 3:**
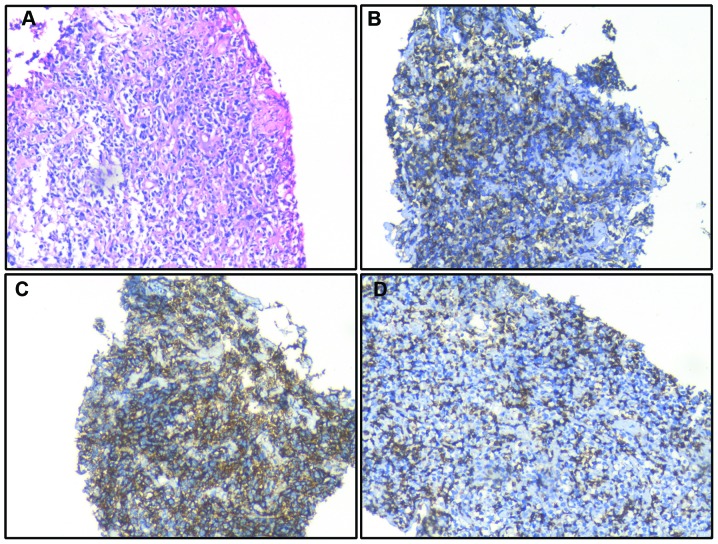
Immunohistochemical examination of the diffuse large B-cell lymphoma (x100). (A) Hematoxylin and eosin staining, and positivity for (B) B-cell lymphoma-2(+), (C) cluster of differentiation (CD)20(++) and (D) CD3(+).

Ultrasound (US) of the prostate revealed large masses measuring 93×79 mm. A pelvic CT scan (Fig. 1A) showed multiple low-density masses, with unclear boundaries with the bladder, prostate and rectum. Three enlarged lymph nodes were observed on the right side of the pelvis, with diameters of 3.5 cm each. The findings met the diagnostic criteria of lymphoma. A bone marrow biopsy showed no abnormalities. Due to significant bleeding, a Foley catheter was inserted and continuous bladder irrigation was commenced. The use of antibiotics and a blood transfusion yielded unsatisfactory results. Due to the rapid loss of blood, the blood pressure decreased resulting in shock and the hemoglobin level decreased to 55 g/l. A bilateral iliac arteriography and chemoembolization (40 mg pirarubicin and 2 mg vincristine) were performed as emergency procedures under local anesthesia (Fig. 2). The duration of surgery was 45 min and the bleeding was stopped 15 min after the surgery. Immunohistochemical examination of a mass demonstrated the following results: Positivity for cluster of differentiation (CD)20(++), CD3(+), leukocyte common antigen(+++), B-cell lymphoma (BCL)-2(+), BCL-6(+), MUM-1(+), vimentin(+) and S-100(+), with a Ki-67 of 80%; and negativity for PSA, human melanoma black 45, P504S, 34βE12, p63, cytokeratin (CK), CK20, villin, caudal-type homeobox-2, smooth muscle actin, myogenin, CD30 and CD10 (Fig. 3). Immunohistochemistry was assessed using the Intensity Reactivity Score (IRS), which evaluates both the staining intensity and the percentage of positive cells. Two pathologists at the Second Xiangya hospital evaluated scores independently. The staining intensity (SI) of the dye color was graded as either : 0, no color; 1, light yellow; 2, light brown; or 3, brown. The reactivity was determined according to the percentage of positive cells (PP) in the total number of cells where there were no negative specimens: 1–10%, 1; 11–30%, 2; 31–50%, 3; 51–80%, 4; and >80%, 5. The IRS was calculated by multiplying the SI with the PP resulting in a minimum score of 0 and a maximum score of 15. Subsequently, the specimens were assigned one of four different levels: 0, (−); 1–4, (+); 5–9, (++); and 10–15, (+++). Combined morphological and immunophenotyping examinations confirmed the diagnosis of a DLBCL of non-germinal center B-cell origin. Subsequent to pathological confirmation of the diagnosis, the patient received intravenous chemotherapy consisting of three courses of a low-dose R-CHOP chemotherapy regimen. This was composed of 375 mg/m^2^ rituximab, 500 mg/m^2^ cyclophosphamide, 20 mg/m^2^ doxorubicin and 1 mg/m^2^ vincristine on day 1 and 50 mg/m^2^ prednisone from days 1–5.

Following one course of chemotherapy, the patient achieved partial remission, and the CT examination showed that the size of the tumor had been reduced significantly (Fig. 1B). Subsequent to three courses of chemotherapy, the patient achieved complete remission. The patient was followed up for eight months, and is currently in a stable condition, with no sign of recurrence of the hematuria.

## Discussion

Primary malignant lymphoma of the prostate, accounting for <0.1% of all non-Hodgkin’s lymphomas, are globally rare. Clinical misdiagnosis of this condition has been common, particularly in the elderly, due to the high similarity with benign prostatic hyperplasia (BPH) or prostate cancer with regard to the symptoms exhibited, including increased urinary frequency, urinary urgency, hematuria and acute urinary retention (5,6). The criteria for a diagnosis of primary prostatic lymphoma are as follows: A tumor only located at the prostate; no lymphoid node or tissue involvement, including the blood vessels; and a lymphoma free period of at least one month after diagnosis (6,7). In the present study, no other lymphoid nodes or tissue involvement was observed in the patient, as confirmed by systemic examination, in accordance to the diagnostic criteria.

Imaging examinations, including CT, transrectal US (TRUS) and positron emission tomography (PET)/CT, are useful in assisting a diagnosis of the primary prostatic lymphoma. Early fluorodeoxyglucose-PET is predictive of prognosis in patients with aggressive NHL, and is useful for selecting patients who could benefit from alternative treatments (6,8,9). The final diagnosis can be established by pathological and immunohistochemical examinations of samples obtained by surgery or transrectal prostate biopsy. In the present study, the CT revealed features that were similar to the published cases of NHL, such as enlargement of the prostate, with low density lesions in the presence or absence of abdominal and pelvic enlargement of the lymph nodes (6,10), which were subsequently confirmed by pathological and immunohistochemical examination.

Due to its rarity, there is no consensus on the preferred treatment for prostatic lymphoma at present. Previous studies demonstrated that R-CHOP chemotherapy was superior to CHOP, a previous standard treatment for NHL (11). The studies suggested that it was the optimal choice for curative intent, and that this combination should become the standard treatment for DLBCL (12–14). Rituxan, a chimeric monoclonal antibody of CD20, a cell-surface protein that is present as one of the typical characteristics of DLBCL, could specifically target the CD20 antigen of B-cell lymphoma. Thus, Rituxan could enhance the cytotoxic sensitivity of lymphoma cells, and synergize with chemotherapy without a clinically significant increase in toxicity (15). In the present study, due to the poor general condition and low hemoglobin levels of the patient, a low-dose R-CHOP chemotherapy regimen was adopted. Following three courses of chemotherapy, the patient achieved complete remission, which suggested that the chemotherapy regimen had exhibited a positive effect. The advantage of low-dose R-CHOP for this patient was that it could minimize the side-effects of chemotherapy, while simultaneously achieving the maximum associated benefits.

The clinical strategies for the treatment of hematuria of prostatic origin consist of medical, surgical and minimally invasive approaches. Conservative means to stop hematuria include the use of 5α-reductase inhibitors and placement of a Foley catheter with continuous bladder irrigation. However, in the case of inoperable patients with acute or hyperacute hematuria, interventional radiology appears to be a suitable alternative option (3). Hald and Mygind (16) first reported unilateral hypogastric artery embolization for the treatment of severe bladder hemorrhage in 1974. Selective arterial prostatic embolization has subsequently been demonstrated to be useful in treating refractory hematuria secondary to prostate cancer or BPH (4). The cessation of bleeding occurs quickly after this procedure and can be subsequently maintained without anesthesia (3). Recently, arterial chemoembolization via various drug carrier systems has become a suitable treatment for localized tumors (17). Combining chemotherapy with embolization may maximize the curative effects. Firstly, embolization could reduce the blood supply of a tumor, thus slowing down the growth of the tumor. Secondly, it could also slow down the local blood circulation, so that chemotherapy drugs can stay longer in the lesions, thus significantly improving their effectiveness (18). In the present study, the patient developed a prostate tumor complicated by an acute massive hemorrhage; conservative treatments, such as placement of a Foley catheter with continuous bladder irrigation and a blood transfusion, were unsuccessful. Considering that the patient’s blood pressure decreased to 80/50 mmHg (normal range, 90/60–140/90 mmHg) and heart rate increased to 100 beats per min from 85 beats per min (normal range, 60–100 beats per min) indicating that the patient was in a state of shock and that they were not suitable for a transurethral resection of the prostate under general anesthesia, bilateral iliac arterial infusion chemotherapy and embolization were chosen, both of which could impair the tumor and quickly stop the bleeding. Surgery effectively stopped the bleeding under local anesthesia and the duration of the procedure was short. Post-operatively, the patient only developed mild hip pain and low thermal discomfort, without serious complications such as a recto-urethral fistula. Therefore, we propose that bilateral iliac arterial infusion chemotherapy and embolization could be a safe and effective method to treat prostate tumor with intractable hemorrhage.

Due to the rarity of primary prostatic lymphoma, its prognosis is unclear. In a retrospective review of 62 patients, Bostwick *et al* (7) demonstrated that the specific five-year survival rate was only 33%, and 73% of patients with primary prostatic lymphoma developed metastasis one to 59 months after diagnosis. However, another review of 23 cases showed that patients responded well to chemotherapy and could possibly be cured if the primary prostatic lymphoma was confined to the prostatic region (19). Serum LDH has proven to be an important prognostic factor for non-Hodgkin’s lymphoma, with an inverse correlation detected between levels of LDH and the survival rate (20). Increased serum LDH levels suggest a poor prognosis. In the present case, the patient was >60 years old with a high LDH, which suggested a poor prognosis. However, in cooperation with the Department of Intervention and Hematology (Second Xiangya Hospital), R-CHOP was administered and interventional embolization was performed on the patient, which achieved a favorable effect. Following three courses of chemotherapy, the patient was followed up for eight months, and currently remains in a stable condition. Further observation is required to obtain long-term results.
